# *In Vivo* Evaluation of the Cardiometabolic Potential of Grape Pomace: Effect of Applying Instant Controlled Pressure Drop

**DOI:** 10.3390/foods11213537

**Published:** 2022-11-07

**Authors:** Yuridia Martínez-Meza, Jara Pérez-Jiménez, Luis Miguel Salgado-Rodríguez, Ana Karen Castellanos-Jiménez, Rosalía Reynoso-Camacho

**Affiliations:** 1 Facultad de Química, Universidad Autónoma de Querétaro, Querétaro 76010, Mexico; 2 Department of Metabolism and Nutrition, Institute of Food Science, Technology and Nutrition (ICTAN-CSIC), José Antonio Novais 10, 28040 Madrid, Spain; 3 CIBER of Diabetes and Associated Metabolic Disease (CIBERDEM), ISCIII, 28029 Madrid, Spain; 4 CICATA-Querétaro, Instituto Politécnico Nacional, Querétaro 76090, Mexico

**Keywords:** grape pomace, instant controlled pressure drop, cardiometabolic effects, insulin resistance, plasma triacylglycerols

## Abstract

Grape pomace (GP) is a source of polyphenols which may be present as free structures or associated with dietary fiber. Instant controlled pressure drop (DIC) is a technology which can modify the association of polyphenols with food matrixes, but how these modifications affect the health benefits associated with GP remains to be elucidated. In this study, in rats fed a high-fat–fructose diet (HFF), we evaluated the *in vivo* cardiometabolic effects of the modification of polyphenols in GP caused by DIC at 0.2 MPa for 60 s (DIC1) and 0.4 MPa for 120 s (DIC2). These treatments increased anthocyanin and total flavonoid contents, respectively, while all the supplementations caused significant improvements in insulin resistance and plasma triacylglycerols. Thus, the bioactive compounds present in GP (including a major fraction of non-extractable proanthocyanidins) caused these modifications independently of the specific polyphenol profiles which may have resulted from these DIC treatments. Additionally, only intact GP led to an increase in HDL cholesterol, while only DIC2-treated GP improved hepatic steatosis. In conclusion, GP always improves insulin sensitivity in this animal model of obesity, while the different compositions of GP modified by DIC may be associated with other cardiometabolic parameters.

## 1. Introduction

Grape pomace (GP) is a by-product of the wine industry. It contains significant amounts of phytochemicals related to health benefits associated with its consumption, such as decrease in weight gain [1], improvement in insulin resistance [1,2,3], decrease in adiposity [2] and attenuation of liver triglyceride accumulation [1,2]. Most studies have been conducted with grape pomace extracts (GPEs), i.e., fractions rich in extractable polyphenols (EPPs), obtained by different chemical extraction procedures. However, important concentrations of polyphenols remain in the extraction residue, and these are known as non-extractable polyphenols (NEPPs) [4]. A comprehensive approach to analyzing the beneficial effects of GP should therefore consider the contributions of both EPPs and NEPPs to the whole profile of phytochemicals present in this material. Moreover, intact NEPPs reach the colon, where they provide a substrate for colonic microbiota [5], as do a fraction of EPPs.

The health benefits of GP are also related to its high dietary fiber (DF) content. A comparative study showed that GP was more effective in preventing increased systolic blood pressure and plasma triacylglycerol levels than GPE, and these differences were associated with DF [2]. Integral GP combines DF, EPPs and NEPPs in a single material, so the health benefits related to its consumption will be dependent on these compounds and their interactions with each other. In addition, GP processing by different techniques may cause modifications to the polysaccharide and polyphenol contents and their profiles, which may affect its health effects. In this regard, implementing methodologies that improve the bioaccessibility of polyphenols in food matrixes constitutes an important research field [6].

Application of an instant controlled pressure drop (DIC—this being its acronym in French—détente instantanée contrôlée) is a thermo-mechanical process that induces expansion of a food matrix through the injection of saturated steam at a fixed pressure, leading to the instant evaporation of water inside the food matrix [7]. It has been used as a strategy for structural modification by increasing the diffusion of solvents within matrixes and therefore phytochemical extraction [8]. We have previously shown that DIC modifies the morphologies and profiles of GP polyphenols, producing phenolic compounds with a simpler structure as a result of the partial depolymerization of proanthocyanidins and anthocyanin degradation. In addition, the hydrolysis of dietary fiber was found to be associated with an increase in soluble DF [9]. These modifications might be associated with different health effects from those of intact GP, since, for instance, it has been reported that a low degree of polymerization of proanthocyanidins is relevant to their ability to inhibit digestive enzymes and could thus affect starch and fat absorption [10]. However, the potential modifications of the health effects related to GP due to the application of DIC have not been assessed. 

The present study was therefore based on the concept that the modifications to EPPs, NEPPs and DF in GP previously reported to be due to DIC application may lead to modifications in the anti-obesogenic effects associated with GP consumption. For this reason, we performed a study of an animal model fed an obesity-inducing diet, in which animals were supplemented with intact GP or GP subjected to different DIC conditions in order to evaluate the effects on several parameters related to obesity. 

## 2. Materials and Methods

### 2.1. Raw Material

Grape pomace (Malbec) was collected after the pressing process from a wine producer (La Redonda) located in Queretaro, Mexico. The samples were stored in hermetic containers at −20 °C, protected from light and oxygen. 

### 2.2. Application of Instant Controlled Pressure Drop (DIC)

The conditions of the DIC process were selected according to previous results [9]. Two treatments resulted in important changes in the contents of phenolic compounds: 0.2 MPa for 60 s (DIC1) produced an increment in anthocyanin contents; 0.4 MPa for 120 s (DIC2) caused the depolymerization of proanthocyanidins and generated phenolic acids as a product of anthocyanin degradation. We used LABIC 0.1 DIC equipment (ABCAR-DIC Process, La Rochelle, France). After DIC application, the samples were collected. The untreated samples and those treated with DIC were all dried on a tray dryer at 45 °C, ground and sieved through a 40-mesh sieve (<425 µm), and stored at −20 °C until analysis.

### 2.3. Polyphenol and Total Dietary Fiber (TDF) Analysis of GP

The polyphenol content of the GP with or without DIC treatment was assessed using spectrophotometry methods; detailed polyphenol compositions determined by advanced analytical techniques have been reported elsewhere [9,11]. Despite the reported limitations of spectrophotometry methods, it should be highlighted that those used here have been validated for highly diverse food matrixes and are widely used in the field. 

For EPP analysis, extracts were obtained by the procedure described by Pérez-Jiménez and Saura-Calixto [12]. Two successive extractions were carried out, the first with methanol:water (50:50, *v*/*v*) acidified with HCl (pH 2) and the second with acetone:water (70:30, *v*/*v*). The total extractable phenolic compounds (TEPCs) were quantified using the Folin–Ciocalteu procedure; a calibration curve was elaborated with gallic acid as a standard [13]. Total monomeric anthocyanin content was determined via the pH differential method [14]. Total flavonoids were determined according to the procedure reported in Morales-Luna et al. [14] and quantified by comparison with a catechin calibration curve. Finally, extractable proanthocyanidins were determined in acetone extracts using the methodology described by Zurita, Díaz-Rubio and Saura-Calixto [15], and we used the calibration curve reported by the same authors.

The residue obtained in the EPP extraction was used to analyze the NEPP fraction, which consisted of hydrolyzable polyphenols (HPPs) and non-extractable proanthocyanidins (NEPAs). The hydrolysis procedure for HPPs and further analyses were performed as reported by Pérez-Jiménez and Saura-Calixto [12], while NEPA depolymerization and analysis followed the protocol described by Zurita, Díaz-Rubio and Saura-Calixto [15]. 

The TDF was determined according to AOAC methods [16], specifically with an enzymatic–gravimetric method using a Total Dietary Fiber Assay Kit (Sigma TDF-100A Kit, Sigma, Saint Louis, MO, USA).

These polyphenol and TDF results were used to calculate the EPP, NEPP and DF contents in the diets consumed, according to the GP added (1 g/kg body weight/day).

### 2.4. Animals and Diets

Seventy male Wistar rats (190 ± 5 g) were obtained from the Institute of Neurobiology, UNAM (Querétaro, México). The ethics committee of the Universidad Autónoma de Queretaro (Queretaro, Mexico) approved the experimental protocol (CBQ 18/010). The animals were subjected to a 12 h dark/light cycle at 25 °C. After acclimatization, the animals were randomized into five experimental groups (n = 14). The healthy control group was fed a powdered standard (STD) diet (Rodent Lab Chow 5001, Purina, Saint Louis, MO, USA). The obese group was fed a high-fat–fructose (HFF) diet consisting of 60% powered STD diet, 20% fat (pork fat) and 20% fructose (by weight), complemented with vitamins and minerals (0.03% to the total weight of the feed). The three treatment groups were fed the HFF diet supplemented with GP (1 g/kg body weight/day); the contents of polyphenols and dietary fiber were calculated using the data obtained, as explained in the previous section. The control group was supplemented with GP without DIC treatment (HFF + GP), the second group was supplemented with GP treated at 0.2 MPa for 60 s (HFF + DIC1) and the third group with GP treated at 0.4 MPa for 120 s (HFF + DIC2). Food and water were administered via autonomous oral feeding (ad libitum), and the animals were weighed weekly. 

To evaluate oxidative states, six animals were slaughtered without fasting conditions, and we obtained serum samples. For feces collection, before being slaughtered, the rats were housed in metabolic cages for 24 h and maintained with food and water ad libitum.

#### 2.4.1. Body Weight Gain

The body weight changes were monitored weekly throughout the experimental period (16 weeks). Weight gain (g) was calculated based on the differences reported weekly, as dt = tw − t0, where tw is the weight reported weekly, and t0 is the initial weight. In addition, food consumption was measured twice a week by weight difference. The amount of food consumed was used to determine EPP, NEPP and DF.

#### 2.4.2. OGTT and Glucose Assay

The animals were subjected to an oral glucose tolerance test (OGTT) after overnight fasting, one week before slaughter. Glucose (2 g/kg) was administered by oral gavage. Drops of blood were drawn from the tail vein at 0 (before glucose administration), 15, 30, 60 and 120 min after glucose loading to evaluate the blood glucose concentrations. Blood glucose was measured using a handheld glucose meter (ACCU-CHEK, Roche, Basel, Switzerland), and the trapezoidal rule was used to calculate areas under the curves.

#### 2.4.3. Slaughter

After sixteen weeks, the animals were euthanized by guillotine decapitation after overnight fasting. Immediately after decapitation, the blood of each animal was collected and centrifuged at 4000× *g* for 15 min to obtain serum. Fat and liver tissues were collected and frozen with liquid nitrogen. Part of the liver tissue was fixed in formaldehyde (10%) for histological analysis.

Visceral adipose tissue (epididymal, retroperitoneal and mesenteric) was excised (separately) and weighed. The adiposity index was calculated as the total adipose tissue weight divided by the total body weight at 16 weeks. 

#### 2.4.4. Evaluation of Oxidative Status

Oxidative status was evaluated in serum and feces, with TEPCs determined by Folin–Ciocalteu assay [17], while the antioxidant capacities were estimated using the ABTS method, which assesses radical scavenging capacity [18]. First, serum samples were deproteinized with an equal volume of acetonitrile and incubated for 2 min at 25 °C. Then, the sera were centrifuged at 4 °C and 9500× *g* for 10 min. The supernatants were recovered to quantify the total polyphenol contents and antioxidant capacities [19].

Fecal extracts were obtained by mixing the product of successive extractions with methanol/water acidified with HCl (50:50, *v/v*; pH 2) and acetone/water (70:30, *v/v*). After centrifugation (15 min, 3000× *g*), the supernatants were combined to determine the polyphenol contents and antioxidant capacities.

For the Folin–Ciocalteu assay, 10 µL of deproteinized serum or fecal polyphenol extract was mixed with 40 µL of distilled water and 25 µL of Folin reagent. Then, 125 µL of Na_2_CO_3_ (5% *m/v*) was added to the mixture, which was then incubated for 30 min, and the absorbance was measured at 765 nm. The results were expressed as mg gallic acid equivalent per mL for serum samples. The scavenging capacity of the 2,2′-azino-bis(3-ethylbenzothiazoline-6-sulfonic acid) (ABTS) free radical was determined for deproteinized serum and fecal polyphenol extracts. The ABTS radical was prepared by mixing ABTS with 2.45 mM potassium persulfate, followed by incubation overnight in darkness. Then, it was diluted in 5 mM phosphate buffer with 0.145 M NaCl adjusted to pH 7.4 to obtain an absorbance of 0.70 ± 0.02 at 730 nm. A quantity of 10 µL of extract was then mixed with 200 µL of the ABTS radical, and absorbance was measured after 6 min at 734 nm; the results were expressed as mmol Trolox equivalents/mL [18] and corresponded to the ABTS values.

#### 2.4.5. Total Proanthocyanidins in Feces

Proanthocyanidin (PA) extraction was carried out with dry feces. Before PA extraction, each sample (0.1 g) was defatted with 8 mL of hexane; this was then stirred for 20 min. Then, the sample was centrifuged at 1800× *g* for 10 min, the supernatant was discarded and the PA content of the residue was determined. The residue was treated with 10 mL of butanol/HCl (97.5:2.5, *v/v*) with an additional 0.1% FeCl_3_. The mixture was incubated in boiling water for 1 h and centrifuged at 2500× *g* for 10 min. The supernatants were recovered and washed twice with 5 mL of HCl/butanol/FeCl_3_, and the final volume was adjusted to 25 mL. Absorbance of the extracts was measured at 555 nm, and the results were expressed as mg eq polymers/g [20].

#### 2.4.6. Insulin Resistance Estimation

Fasting insulin in serum was quantified using an enzyme-linked immunosorbent kit (ELISA) (Millipore, Burlington, MA, USA). Serum glucose was quantified using an enzymatic colorimetric kit (SpinReact, Sant Esteve de Bas, Spain). Homeostasis model assessment (HOMA-IR) was used to estimate insulin resistance using the following equation:

HOMA-IR = fasting insulin (μU/mL) × [fasting glucose (mmol/L)/22.5].

#### 2.4.7. Quantification of Total Cholesterol, HDL-c and Triacylglycerols

In serum, total cholesterol, triacylglycerols and HDL-c were quantified using an enzymatic colorimetric kit (SpinReact, Sant Esteve de Bas, Spain). 

#### 2.4.8. Histological Analysis of Liver and Hepatic Triacylglycerol Contents

For histological micrographs, each liver tissue sample was placed in 10% formalin. Afterwards, the tissues were fixed in paraffin and sectioned into 5 μm sheets to be stained in a hematoxylin and eosin solution. Finally, photographs were taken at 40× magnification. 

The extraction of triacylglycerols from the liver was performed following Schlezingeret al. [21]: 200 mg of tissue was homogenized and 500 µL of 30% KOH/ethanol (2:1 *v/v*) was added. Subsequently, the sample was incubated for 12 h at 55 °C, and ethanol/water (1:1 *v/v*) was added to a final volume of 1000 µL. Then, the sample was centrifuged at 10,000× *g* for 5 min, at 4 °C, and the supernatant was recovered and brought to a final volume of 1200 µL with ethanol/water (1:1 *v/v*). An aliquot of 200 µL was mixed with 215 µL of MgCl_2_ for 10 min. The sample was centrifuged at 10,000× *g* for 5 min, and the supernatants were used to quantify triacylglycerols using an enzymatic kit. 

### 2.5. Statistical Analysis

The results are expressed as mean values ± standard deviations. The data were analyzed using the Shapiro–Wilk test to determine normal distributions. Means were compared using one-way analysis of variance (ANOVA). The parametric data were analyzed with Tukey’s test, and the significance level was set at α < 0.05. Statistical analyses were performed using JMP software 11.0 version (SAS Institute, Cary, NC, USA). 

## 3. Results and Discussion

### 3.1. Polyphenol Intake in a HFF Diet Supplemented with GP Subjected to DIC

Total polyphenol intake (considering both EPP and NEPP fractions) was determined based on the amount of food consumed by animals fed a HFF diet supplemented with 1 g/kg/day of GP. As expected, supplementation with GP, with or without DIC, significantly increased polyphenol intake in all classes (Table 1). Nevertheless, some significant differences were observed between the different supplemented groups regarding extractable flavonoids, monomeric anthocyanins and NEPA intake. Thus, in both HFF and HFF + DIC1 there was a high NEPA intake, which was significantly lower in HFF + DIC2. In contrast, the HFF + DIC2 group showed significantly higher extractable flavonoid consumption than the HFF + GP and HFF + DIC1 groups. In addition, HFF + DIC1 was the group with the highest monomeric anthocyanin intake. At the same time, all the GP groups showed significantly higher total DF intakes. These differential values were the result of the changes in GP composition caused by DIC [9] i.e., depolymerization of NEPAs. 

### 3.2. Effect of GP Treated with DIC on Antioxidant Status

Total polyphenol contents and antioxidant capacities of the plasma and feces of the rats were measured in order to evaluate modifications in antioxidant status caused by GP supplementation, with or without DIC treatment. Additionally, total PAs were also measured in feces, as characteristic polyphenols found in GP which are known to be modified by DIC [9].

Regarding the serum results (Table 2), all the HFF groups exhibited higher total phenolic compound (TPC) and ABTS values than the STD group; the former increased by 4.4%, 30.5%, 28.4% and 38.4% in HFF, HFF + GP, HFF + DIC1 and HFF + DIC2, respectively. For ABTS, these increases were 9.3%, 5.4%, 12% and 27.6%, respectively, and may have been associated with the interferences known to characterize these methods [22]. Nevertheless, significant increases in both TPC and ABTS values were observed in the HFF + DIC2 group, which was the group with the highest extractable flavonoid intake, suggesting a specific contribution of these polyphenols to the determinations. 

Regarding the fecal samples, supplementing the HFF diet with grape pomace subjected or not to DIC treatment significantly increased TPCs, total proanthocyanidins (TPAs) and antioxidant capacities as compared to the HFF group. Among the three GP groups, the HFF + GP group exhibited the highest TPA content, while HFF + DIC1 and HFF + DIC2 had the highest TPC and antioxidant capacity values, without significant differences between the two groups. The fact that the highest TPA concentration was found in the HFF + GP group may have been due to the greater presence of high-molecular-weight PAs in the feed provided to these animals as compared to the GP given to DIC1 and DIC2, as previously reported [9]. Indeed, it has been suggested that total PA determination in feces may be used as a biomarker of the intake of these compounds after supplementation with them [20]. It should be highlighted that the higher polyphenol contents and antioxidant capacities in the fecal samples of the supplemented groups have biological relevance, as they indicate that the supplementation contributed to an antioxidant environment in the colon. Furthermore, a high fecal polyphenol concentration is associated with high polyphenol circulation [23], which should be assessed by specific metabolite analysis. 

### 3.3. Effect of GP Treated with DIC on Weight Gain and Fat Accumulation

**Figure 1:**
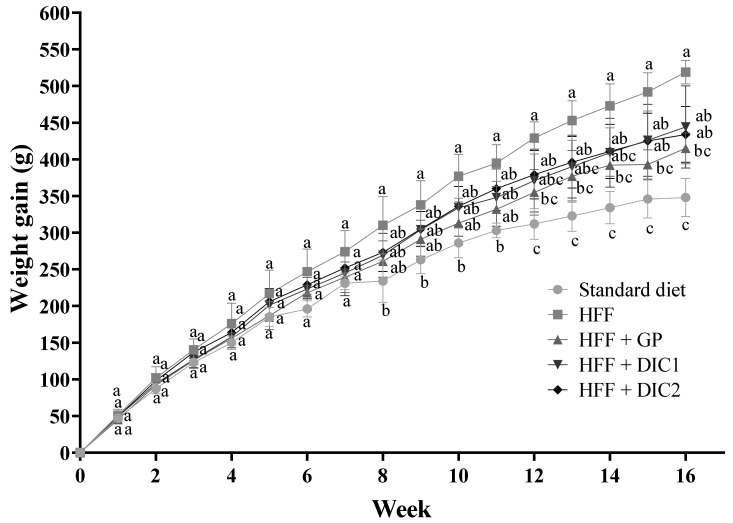
Body weight gain of animals fed a HFF diet supplemented or not with grape pomace subjected or not to DIC. Different letters indicate significant differences between the groups. Data are reported as means ± SEs for eight animals per group. Statistically significant differences were determined by ANOVA, followed by Tukey’s test (*p* < 0.05). GP, grape pomace without DIC; DIC1, grape pomace with DIC at 0.2 MPa for 60 s; DIC2, grape pomace with DIC at 0.4 MPa for 120 s.

The evolution of body weight gain in the different experimental groups is shown in Figure 1. The difference in body weight gain between the obese (HFF) and healthy (STD) control groups was significant from the eighth week on (Figure 1). Thus, the average body weight gain was 519 ± 16 g for the HFF group and 348 ± 26 g for the healthy STD group at the end of the experimental period. The groups fed the HFF diet supplemented with GP showed less weight gain than the HFF group: 20% less for the HFF + GP group, 14% for HFF + DIC1 and 16% for HFF + DIC2. This reduction was statistically significant for the HFF + GP group as compared to the HFF group, but in the case of HFF + DIC1 and HFF + DIC2 it did not reach statistical significance. Nevertheless, the observed lower rate of increase may potentially have a health impact; a decrease in body weight of 5–10% has been reported [24] to be associated with preventing obesity-related metabolic alterations. 

Regarding adiposity, we observed that there was more white adipose tissue (WAT), comprising epididymal, retroperitoneal and mesenteric WAT, in the animals fed the HFF diet than in the STD group (Table 3). All the GP supplemented groups exhibited a significant decrease in mesenteric fat and adiposity index compared to the HFF group, without significant differences between them. This agrees with the reports that some flavonoids, such as catechin or epicatechin (structural units of proanthocyanidins), are the main components of GPEs and can suppress weight gain and adipose tissue [25]. In contrast, GP supplementation was not capable of reverting the increase in epididymal fat caused by the HFF diet. In the case of mesenteric fat, both the HFF + GP and HFF + DIC2 groups experienced a significant decrease compared to the HFF group, which was not observed in the HFF + DIC1 group. Since this last group had the highest anthocyanin consumption, this effect may have been associated with the low bioavailability reported for this polyphenol class [26].

### 3.4. Effect of GP Treated with DIC on Glucose Homeostasis and Insulin Resistance

**Figure 2:**
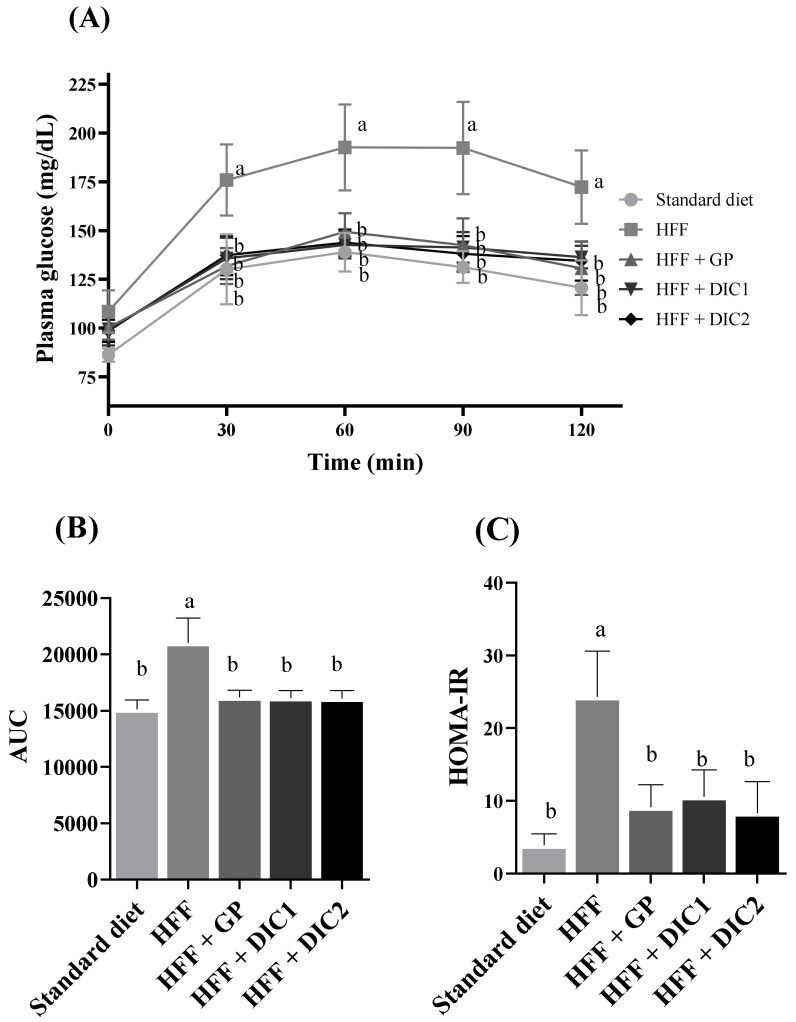
Effects of grape pomace subjected or not to DIC on glucose homeostasis in rats fed a HFF diet: (**A**) plasma glucose levels (mg/dL); (**B**) mean areas under the curves (AUCs) measured between 0 and 120 min after glucose loading; and (**C**) HOMA indexes. Data are reported as means ± SEs for eight animals per group. Different letters indicate significant differences between the groups. Statistically significant differences were determined by ANOVA, followed by Tukey’s test (*p* < 0.05). GP, grape pomace without DIC; DIC1, grape pomace with DIC at 0.2 MPa for 60 s; DIC2, grape pomace with DIC at 0.4 MPa for 120 s.

GP consumption has been associated with a beneficial effect on glucose homeostasis via improved glucose tolerance and lower insulin resistance [1]. This was also observed in our study, as evidenced by the results for glucose levels in the OGTT (Figure 2A). Thus, fasting and postprandial glucose levels at the different sampling times were significantly higher for the HFF group, showing that the diet-induced obesity model also generated hyperglycemia, while similar results were obtained for the STD group and all the GP groups. In addition, significantly lower AUC values were obtained for the treated groups (HFF + GP, HFF + DIC1 and HFF + DIC2), which were similar to those for the STD group (Figure 2B). This indicates that GP, treated or not with DIC, improved glucose intolerance. 

The hypoglycemic effect has been attributed to the proanthocyanidins in grape seeds through the inhibition of DPP-IV (dipeptidyl peptidase-IV), which generates an increase in the activity of intestinal incretin [27] and inhibition of α-glucosidases and pancreatic α-amylase activities in the small intestinal endothelium [28]. Table 1 shows that the total proanthocyanidins ingested (extractable and non-extractable) were 150, 145 and 125 mg/kg body weight/day for the HFF + GP, HFF + DIC1 and HFF + DIC2 groups, respectively. Although the HFF + DIC2 group consumed the least proanthocyanidins, compared with other studies, the dose was sufficient to show glucose homeostasis regulation. For example, Wistar rats fed a dose of 100 mg/kg body weight/day of proanthocyanidin extract from grape seed showed reductions in glucose and insulin levels [29]. Interestingly, most PAs in GP are NEPAs, commonly ignored in studies of polyphenols but which are associated with several health-related properties [5,30].

Regarding the HOMA-IR index (the model used to determine the relationship between glucose and insulin to assess insulin sensitivity), the same trend was observed as for glucose (Figure 2C). Thus, the HOMA-IR value for the HFF group was 6.2 times higher than that for the group fed the STD diet, showing the adequacy of the animal model for inducing insulin resistance. At the same time, the HOMA-IR values of the animals fed the HFF diet supplemented with GP (with or without DIC) did not present any significant statistical differences and were similar to those of the healthy STD group. The increase in insulin signaling brought about by grape seed proanthocyanidins has been associated with activation of the PI3K pathway and the promotion of insulin action by reduced serine kinase activation and cytokine signaling [29]. This improvement has also been associated with the main flavonoids found in grapes: (−)-epicatechin, (+)-catechin and quercetin [31]. 

Therefore, the improvement in glucose homeostasis and insulin resistance was similar for all the GP groups, independently of DIC application, and supplementation restored the original values found in the STD group. Since intact GP already exhibits the capacity to improve insulin response, as also evidenced in human studies [32], it seems that the modifications to phenolic profiles caused by DIC do not affect this particular feature of GP. 

### 3.5. Effect of GP Treated with DIC on Serum Lipid Profiles and Hepatic Steatosis

The effects of GP on total cholesterol, triacylglycerols, HDL-c and non-HDL-c are shown in Table 4. Total cholesterol was not modified from the STD control values in the HFF group, but this diet caused a significant increase in triacylglycerols and non-HDL-c levels, together with a significant decrease in HDL-c. Supplementation with GP (with or without DIC treatment) significantly reduced serum triacylglycerols as compared with the HFF group, and in the case of HFF + GP and HFF + DIC2 the values were statistically similar to those of the STD group. Regarding non-HDL cholesterol, all the GP groups exhibited intermediate values between STD and HFF (without significant differences between any of them). Furthermore, HFF + GP showed a significant increase in HDL cholesterol compared to HFF, which was not observed in the GP groups where DIC was applied.

The effects of grape polyphenols on lipid metabolism remain controversial. The mechanism by which they may positively modify lipid profiles may be associated with the flavonoid fraction and their capacity to reduce the absorption of dietary fat in the intestine, decreasing postprandial chylomicron concentrations [33,34]. It has also been reported that proanthocyanidins inhibit hepatic VLDL secretion, reduce plasma lipids and increase beta-oxidation [35]. In our study, the two groups whose lipid profiles were modulated the most were HFF + GP and HFF + DIC2, characterized by higher proanthocyanidin and total flavonoid contents, respectively. Nevertheless, we cannot rule out that the activity of GP was due to its higher DF content, since this constituent has also been associated with a reduction in intestinal absorption of triacylglycerols and increase in their excretion [2]. 

The HDL-c increase, found only in the HFF + GP group, was not observed in other studies with grape pomace [32,36,37]. However, a polyphenol with its highest concentration in GP before DIC treatment [9], kaempferol, has been associated with increased plasma HDL cholesterol concentrations in rats fed a high-fat diet [38].

**Figure 3:**
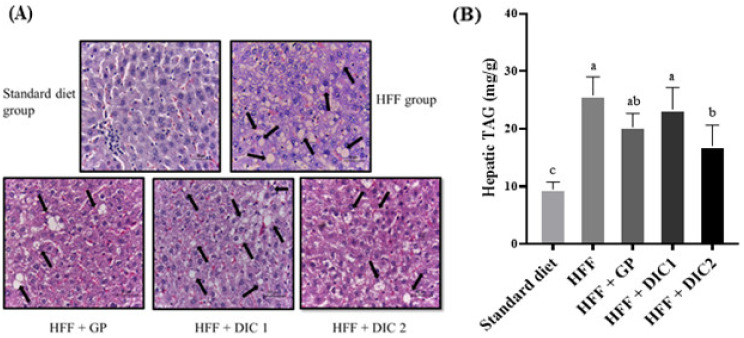
(**A**) Representative liver histological micrographs. (**B**) Hepatic triacylglycerols (mg/dL) of rats fed a high-fat–fructose (HFF) diet supplemented or not with grape pomace subjected or not to DIC. Data are reported as means ± SEs for eight animals per group. Different letters indicate significant differences between the groups. Statistically significant differences were determined by ANOVA, followed by Tukey’s test (*p* < 0.05). GP, grape pomace without DIC; DIC1, grape pomace with DIC at 0.2 MPa for 60 s; DIC2, grape pomace with DIC at 0.4 MPa for 120 s.

Hepatic steatosis is the accumulation of triacylglycerols in hepatic tissue, a common complication associated with obesity. Figure 3A shows the histological analysis of the animals fed the HFF diet and GP treated or not with DIC. According to the histology, the HFF group showed an increase in hepatic lipid deposits compared to the STD group. Regarding the treated groups, morphological analysis of the liver indicated that the DIC2 treatment markedly decreased lipid accumulation, indicated by decreases in hepatic lipid droplets compared to the HFF control group. Hepatic triacylglycerol determinations confirmed this (Figure 3B). Thus, the HFF diet increased triacylglycerol contents by about 2.7-fold compared to the healthy STD group, and only the HFF + DIC2 group had values that were statistically different from those of the obese HFF group (54% lower). These results may be explained by the previous finding that a GP extract was associated with significantly decreased levels of hepatic fatty-acid-synthesis-related enzymes, such as fatty acid synthase, glucose-6-phosphate dehydrogenase and malic enzyme [37]. We should also highlight that the GP group showed values of liver triacylglycerols that were intermediate between those of the HFF and the HFF + DIC2 groups, while those of HFF + DIC1 remained similar to those of the HFF group. Thus, it seems that an increase in anthocyanin content (the main characteristic of the HFF + DIC1 diet) is not associated with an improvement in hepatic steatosis.

## 4. Conclusions

After supplementing animals fed a HFF diet with GP either not subjected to DIC treatment (rich in NEPAs) or subjected to treatment DIC1 (rich in anthocyanins) or treatment DIC2 (rich in flavonoids), we observed that all these supplementations caused significant improvements in insulin resistance and plasma triacylglycerol levels. Thus, the concentrations of bioactive compounds present in GP (including a major fraction of NEPAs) are capable of causing these modifications independently of the specific polyphenol profile that results from the DIC treatment. Additionally, only intact GP increased HDL-c, while only DIC2 improved hepatic steatosis. Thus, it seems that anthocyanin content is not the main characteristic of GP that contributes to its obesity-related effects, although the specific contributions of its other constituents need further research for us to gain a better understanding of them. 

## Figures and Tables

**Table 1:** Extractable and non-extractable polyphenols and dietary fiber in the diets consumed by the rats fed a HFF diet supplemented or not with grape pomace subjected or not to DIC.

	HFF	HFF + GP	HFF + DIC1	HFF + DIC2
EPPs				
TEPC consumption	121.4 ± 2.3 ^c^	188.2 ± 2.9 ^b^	186.6 ± 5.8 ^b^	195.6 ± 2.9 ^a^
EPA consumption	34.1 ± 0.7 ^c^	39.0 ± 0.7 ^b^	39.4 ± 0.9 ^b^	40.1 ± 0.7 ^a^
FV consumption	9.44 ± 0.2 ^d^	54.5 ± 0.6 ^b^	49.3 ± 2.3 ^c^	62.5 ± 0.6 ^a^
MA consumption	—	5.38 ± 0.1 ^b^	6.5 ± 0.4 ^a^	3.6 ± 0.1 ^c^
NEPPs				
HPP consumption	243.1 ± 4.7 ^b^	265.6 ± 0.2 ^a^	267.8 ± 1.3 ^a^	266.5 ± 0.2 ^a^
NEPA consumption	31.0 ± 0.6 ^c^	111.1 ± 1.4 ^a^	105.9 ± 4.6 ^a^	84.9 ± 1.1 ^b^
TDF	8507.7 ± 162 ^b^	9226.3 ± 169 ^a^	9172.8 ± 198 ^a^	9153.9 ± 168 ^a^

Results are expressed as mg/kg body weight/day and as means ± standard deviations. Values in the same row with different superscript letters differ significantly according to Tukey’s test (*p* < 0.05). EPPs, extractable polyphenols; TEPC, total extractable phenolic compound; EPA, extractable proanthocyanidin; FV, flavonoid; MA, monomeric anthocyanin; NEPPs, non-extractable polyphenols; HPP, hydrolyzable polyphenol; NEPA, non-extractable proanthocyanidin; TDF, total dietary fiber.

**Table 2:** Phenolic compounds and antioxidant capacities in feces and sera of rats fed a HFF diet supplemented or not with grape pomace subjected or not to DIC.

	Standard Diet	HFF	HFF + GP	HFF + DIC1	HFF + DIC2
Serum					
ABTS ^1^	31.26 ± 4.41 ^c^	32.65 ± 5.36 ^bc^	40.82 ± 3.25 ^ab^	40.14 ± 1.73 ^ab^	43.27 ± 2.56 ^a^
TPCs ^2^	51.91 ± 7.70 ^b^	56.78 ± 4.50 ^ab^	54.76 ± 6.68 ^ab^	58.15 ± 4.70 ^ab^	66.24 ± 7.75 ^a^
Feces					
ABTS ^1^	25.27 ± 2.91 ^c^	25.20 ± 1.52 ^c^	30.38 ± 5.51 ^b^	40.61 ± 1.82 ^a^	40.22 ± 2.61 ^a^
TPCs ^3^	6.50 ± 0.80 ^c^	6.08 ± 1.13 ^c^	8.52 ± 0.46 ^b^	9.59 ± 0.60 ^a^	9.33 ± 0.34 ^a^
TPAs ^4^	2.54 ± 0.25 ^c^	2.99 ± 0.27 ^c^	14.26 ± 3.00 ^a^	11.28 ± 2.42 ^b^	10.71 ± 2.09 ^b^

Results are presented as the averages of eight independent determinations ± SEs. Values in the same row with different superscript letters differ significantly according to Tukey’s test (*p* < 0.05). ^1^ mmol Trolox equivalents/mL. ^2^ mg GAE/mL. ^3^ mg GAE/100 g. ^4^ mg eq polymers/g. TPCs, total phenolic compounds; TPAs, total proanthocyanidins; GP, grape pomace without DIC; DIC1, grape pomace with DIC at 0.2 MPa for 60 s; DIC2, grape pomace with DIC at 0.4 MPa for 120 s.

**Table 3:** Distribution of adipose tissue of rats fed a HFF diet supplemented or not with grape pomace subjected or not to DIC.

	Standard Diet	HFF	HFF + GP	HFF + DIC1	HFF + DIC2
Epididymal fat (%)	1.51 ± 0.07 ^b^	3.70 ± 0.28 ^a^	3.11 ± 0.37 ^a^	3.37 ± 0.40 ^a^	3.15 ± 0.30 ^a^
Retroperitoneal fat (%)	1.21 ± 0.36 ^c^	4.41 ± 0.16 ^a^	3.27 ± 0.48 ^b^	4.27 ± 0.29 ^a^	3.15 ± 0.22 ^b^
Mesenteric fat (%)	0.80 ± 0.04 ^c^	2.01 ± 0.20 ^a^	1.44 ± 0.02 ^b^	1.91 ± 0.13 ^a^	1.26 ± 0.10 ^b^
Adiposity index	4.08 ± 0.12 ^c^	10.74 ± 0.80 ^a^	8.86 ± 0.60 ^b^	9.31 ± 0.71 ^b^	8.32 ± 0.43 ^b^

Results are presented as averages ± SDs, n = 8. Values in the same row with different superscript letters differ significantly according to Tukey’s test (*p* < 0.05). GP, grape pomace without DIC; DIC1, grape pomace with DIC at 0.2 MPa for 60 s; DIC2, grape pomace with DIC at 0.4 MPa for 120 s.

**Table 4:** Lipid profiles for sera of rats fed a HFF diet supplemented or not with grape pomace subjected or not to DIC.

	Standard Diet	HFF	HFF + GP	HFF + DIC1	HFF + DIC2
Total cholesterol	64.64 ± 8.98 ^a^	69.79 ± 3.42 ^a^	69.83 ± 12.49 ^a^	64.39 ± 9.31 ^a^	69.35 ± 7.56 ^a^
Triacylglycerols	58.57 ± 6.97 ^c^	157.52 ± 10.85 ^a^	75.66 ± 12.33 ^bc^	95.09 ± 6.33 ^b^	79.57 ± 11.17 ^bc^
HDL-c	42.62 ± 2.13 ^a^	32.53 ± 12.40 ^b^	40.56 ± 3.56 ^a^	27.44 ± 2.22 ^b^	28.29 ± 7.88 ^b^
Non-HDL-c	21.44 ± 11.93 ^b^	37.26 ± 4.67 ^a^	30.00 ± 9.96 ^ab^	34.11 ± 8.83 ^ab^	36.70 ± 3.21 ^ab^

Results are presented as the averages (mg/dL) ± SEs. Values in the same row with different superscript letters differ significantly according to Tukey’s test (*p* < 0.05). GP, grape pomace without DIC; DIC1, grape pomace with DIC at 0.2 MPa for 60 s; DIC2, grape pomace with DIC at 0.4 MPa for 120 s.

## Data Availability

Data is contained within the article.

## References

[1] Van Hul M., Geurts L., Plovier H., Druart C., Everard A., Ståhlman M., Rhimi M., Chira K., Teissedre P.L., Delzenne N.M. (2018). Reduced obesity, diabetes, and steatosis upon cinnamon and grape pomace are associated with changes in gut microbiota and markers of gut barrier. Am. J. Physiol. Endocrinol. Metab..

[2] Rodriguez-Lanzi C.R., Perdicaro D.J., Antoniolli A., Fontana A.R., Miatello R.M., Bottini R., Prieto M.A.V. (2016). Grape pomace and grape pomace extract improve insulin signaling in high-fat-fructose fed rat-induced metabolic syndrome. Food Funct..

[3] Costabile G., Vitale M., Luongo D., Naviglio D., Vetrani C., Ciciola P., Tura A., Castello F., Mena P., Del Rio D. (2019). Grape pomace polyphenols improve insulin response to a STD meal in healthy individuals: A pilot study. Clin. Nutr..

[4] Saura-Calixto F. (2012). Concept and health-related properties of nonextractable polyphenols: The missing dietary polyphenols. J. Agric. Food Chem..

[5] Pérez-Jiménez J., Díaz-Rubio M.E., Saura-Calixto F. (2013). Non-extractable polyphenols, a major dietary antioxidant: Occurrence, metabolic fate and health effects. Nutr. Res. Rev..

[6] Ribas-Agustí A., Martín-Belloso O., Soliva-Fortuny R., Elez-Martínez P. (2018). Food processing strategies to enhance phenolic compounds bioaccessibility and bioavailability in plant-based foods. Crit. Rev. Food Sci. Nutr..

[7] Hamoud-Agha M.M., Allaf K., Socaci S.A., Aussenac T., Jean-Claude L. (2019). Instant Controlled Pressure Drop (DIC) Technology in Food Preservation: Fundamental and Industrial Applications. Food Preservation and Waste Exploitation.

[8] Allaf T., Tomao V., Ruiz K., Chemat F. (2013). Instant controlled pressure drop technology and ultrasound assisted extraction for sequential extraction of essential oil and antioxidants. Ultrason. Sonochem..

[9] Martínez-Meza Y., Pérez-Jiménez J., Rocha-Guzmán N.E., Rodríguez-García M.E., Alonzo-Macías M., Reynoso-Camacho R. (2021). Modification on the polyphenols and dietary fiber content of grape pomace by instant controlled pressure drop. Food Chem..

[10] Salvadó M.J., Casanova E., Fernández-Iglesias A., Arola L., Bladé C. (2015). Roles of proanthocyanidin rich extracts in obesity. Food Funct..

[11] Martínez-Meza Y., Pérez-Jiménez J., Castaño-Tostado E., Pérez-Ramírez I.F., Alonzo-Macías M., Reynoso-Camacho R. (2021). Instant Controlled Pressure Drop as a Strategy to Modify Extractable and Non-extractable Phenolic Compounds: A Study in Different Grape Pomace Materials. J. Agric. Food Chem..

[12] Pérez-Jiménez J., Saura-Calixto F. (2018). Fruit peels as sources of non-extractable polyphenols or macromolecular antioxidants: Analysis and nutritional implications. Food Res. Int..

[13] Ranjbar N., Eikani M.H., Javanmard M., Golmohammad F. (2016). Impact of instant controlled pressure drop on phenolic compounds extraction from pomegranate peel. Innov. Food Sci. Emerg. Technol..

[14] Morales-Luna E., Pérez-Ramírez I.F., Salgado L.M., Castaño-Tostado E., Gómez-Aldapa C.A., Reynoso-Camacho R. (2019). The main beneficial effect of roselle (*Hibiscus sabdariffa*) on obesity is not only related to its anthocyanin content. J. Sci. Food Agric..

[15] Zurita J., Díaz-Rubio M.E., Saura-Calixto F. (2012). Improved procedure to determine non-extractable polymeric proanthocyanidins in plant foods. Int. J. Food Sci. Nutr..

[16] Latimer G.W. (2012). Official Methods of Analysis of the Association of Official Analytical Chemists (AOAC).

[17] Lamuela-Raventós R.M., Apak R., Capanoglu E., Shahidi F. (2018). Folin–Ciocalteu Method for the Measurement of Total Phenolic Content and Antioxidant Capacity. Measurement of Antioxidant Activity & Capacity: Recent Trends and Applications.

[18] Mareček V., Mikyška A., Hampel D., Čejka P., Neuwirthová J., Malachová A., Cerkal R. (2017). ABTS and DPPH methods as a tool for studying antioxidant capacity of spring barley and malt. J. Cereal Sci..

[19] Chrzczanowicz J., Gawron A., Zwolinska A., de Graft-Johnson J., Krajewski W., Krol M., Markowski J., Kostka T., Nowak D. (2008). Simple method for determining human serum 2, 2-diphenyl-1-picryl-hydrazyl (DPPH) radical scavenging activity–possible application in clinical studies on dietary antioxidants. Clin. Chem. Lab. Med..

[20] Magdaleno-Tapia C., Quifer-Rada P., Rodríguez-Rodríguez E., Estévez-Santiago R., Waterhouse A.L., Lamuela-Reventós R.M., Olmedilla-Alonso B., Pérez-Jiménez J. (2021). Evaluation of the potential of total proanthocyanidin content in feces as an intake biomarker. Food Res. Int..

[21] Schlezinger J.J., Hyötyläinen T., Sinioja T., Boston C., Puckett H., Oliver J., Heiger-Bernays W., Webster T.F. (2021). Perfluorooctanoic acid induces liver and serum dyslipidemia in humanized pparα mice fed an american diet. Toxicol. Appl. Pharmaco..

[22] Munteanu I.G., Apetrei C. (2021). Analytical methods used in determining antioxidant activity: A review. Int. J. Mol. Sci..

[23] Castello F., Costabile G., Bresciani L., Tassotti M., Naviglio D., Luongo D., Ciciola P., Vitale M., Vetrani C., Galaverna G. (2018). Bioavailability and pharmacokinetic profile of grape pomace phenolic compounds in humans. Arch. Biochem. Biophys..

[24] Ryan D.H., Yockey S.R. (2017). Weight loss and improvement in comorbidity: Differences at 5%, 10%, 15%, and over. Curr. Obes. Rep..

[25] Ohyama K., Furuta C., Nogusa Y., Nomura K., Miwa T., Suzuki K. (2011). Catechin-rich grape seed extract supplementation attenuates diet-induced obesity in C57BL/6J mice. Ann. Nutr. Metab..

[26] Shivashankara K.S., Acharya S.N. (2010). Bioavailability of dietary polyphenols and the cardiovascular diseases. Open Nutraceuticals J..

[27] Sulaiman A.A. (2014). Effect of single oral dose of proanthocyanidin on postprandial hyperglycemia in healthy rats: A comparative study with sitagliptin. J. Intercult. Ethnopharmacol..

[28] Cisneros-Yupanqui M., Lante A., Mihaylova D., Krastanov A.I., Rizzi C. (2022). The α-Amylase and α-Glucosidase Inhibition Capacity of Grape Pomace: A Review. Food Bioproc. Technol..

[29] Yogalakshmi B., Bhuvaneswari S., Sreeja S., Anuradha C.V. (2014). Grape seed proanthocyanidins and metformin act by different mechanisms to promote insulin signaling in rats fed high calorie diet. J. Cell Commun. Signal..

[30] González-Sarrías A., Espín J.C., Tomás-Barberán F.A. (2017). Non-extractable polyphenols produce gut microbiota metabolites that persist in circulation and show anti-inflammatory and free radical-scavenging effects. Trends Food Sci. Technol..

[31] Lanzi C.R., Perdicaro D.J., Landa M.S., Fontana A., Antoniolli A., Miatello R.M., Oteiza P.I., Prieto M.A.V. (2018). Grape pomace extract induced beige cells in white adipose tissue from rats and in 3T3-L1 adipocytes. J. Nutr. Biochem..

[32] Martínez-Maqueda D., Zapatera B., Gallego-Narbón A., Vaquero P., Saura-Calixto F., Pérez-Jiménez J. (2018). A 6-weeks supplementation with grape pomace to subjects at cardiometabolic risk ameliorates insulin sensitivity, without affecting other metabolic syndrome markers. Food Funct..

[33] Ishimoto E.Y., Vicente S.J.V., Cruz R.J., Torres E.A.F.D.S. (2020). Hypolipidemic and antioxidant effects of grape processing by-products in high-fat/cholesterol diet-induced hyperlipidemic hamsters. Food Sci. Technol..

[34] Lupoli R., Ciciola P., Costabile G., Giacco R., Minno M.N.D.D., Capaldo B. (2020). Impact of Grape Products on Lipid Profile: A Meta-Analysis of Randomized Controlled Studies. J. Clin. Med..

[35] Nie Y., Stürzenbaum S.R. (2019). Proanthocyanidins of natural origin: Molecular mechanisms and implications for lipid disorder and aging-associated diseases. Adv. Nutr..

[36] Mellen P.B., Daniel K.R., Brosnihan K.B., Hansen K.J., Herrington D.M. (2010). Effect of muscadine grape seed supplementation on vascular function in subjects with or at risk for cardiovascular disease: A randomized crossover trial. J. Am. Coll. Nutr..

[37] Han H.J., Jung U.J., Kim H.J., Cho S.J., Kim A.H., Han Y., Choi M.S. (2016). Combined supplementation with grape pomace and omija fruit ethanol extracts dose-dependently improves body composition, plasma lipid profiles, inflammatory status, and antioxidant capacity in overweight and obese subjects. J. Med. Food..

[38] Ochiai A., Othman M.B., Sakamoto K. (2021). Kaempferol ameliorates symptoms of metabolic syndrome by improving blood lipid profile and glucose tolerance. Biosci. Biotechnol. Biochem..

